# ATP-Sensitive Potassium Channel Currents in Eccentrically Hypertrophied Cardiac Myocytes of Volume-Overloaded Rats

**DOI:** 10.1155/2011/838951

**Published:** 2011-08-10

**Authors:** Zikiar V. Alvin, Richard M. Millis, Wissam Hajj-Mousssa, Georges E. Haddad

**Affiliations:** Department of Physiology & Biophysics, College of Medicine, Howard University, Washington, DC 20059, USA

## Abstract

ATP-sensitive potassium channels (K_ATP_) protect the myocardium from hypertrophy induced by pressure-overloading. In this study, we determined the effects of these channels in volume-overloading. We compared the effects of a K_ATP_ agonist and a K_ATP_ antagonist on sarcolemmal transmembrane current density (pA/pF) clamped at 20 mV increments of membrane potential from −80 to +40 mV in ventricular cardiac myocytes. The basal outward potassium pA/pF in myocytes of volume-overloaded animals was significantly smaller than that in the myocytes of sham-operated controls. Treatment of the control myocytes with the K_ATP_ agonist cromakalim increased pA/pF significantly. This increase was blocked by the K_ATP_ antagonist glibenclamide. Treatment of the hypertrophied myocytes from volume-overloaded animals with cromakalim and in the presence and absence of glibenclamide did not change pA/pF significantly. These findings suggest that eccentrically hypertrophied cardiac myocytes from volume-overloading may be unresponsive to specific activation/inactivation of K_ATP_ and that dysfunctional K_ATP_ may fail to protect the myocardium from left ventricular hypertrophy associated with volume-overloading.

## 1. Introduction

Ventricular hypertrophy is an adaptation to a wide variety of cardiac insults such as hypertension, myocardial infarction, and valvular heart diseases [1]. Patients diagnosed with aortic regurgitation are shown to be at greater risk for complications associated with heart failure, valve replacement surgeries, and sudden death than patients with aortic stenosis [2]. ATP-sensitive potassium channels (K_ATP_) are thought to provide mechanisms for adaptation of cardiac myocytes to hypoxia, ischemia, oxidative stress, and hypertrophy [3]. The opening of sarcolemmal K_ATP_ is reported to decrease the duration of action potentials which may conserve ATP stores [4]. Intermittent hypoxia-induced opening of sarcolemmal and mitochondrial K_ATP_ is reported to precondition the heart and ameliorate the adverse effects of ischemic insults [5]. Inactivating mitochondrial K_ATP_ is shown to antagonize this ischemic preconditioning [6]. Inactivating sarcolemmal K_ATP_ may also attenuate sympathetic signal transduction in cardiac sympathetic ganglia [7], contract coronary arterial smooth muscle, and increase susceptibility to vasospasm [8] and cardiac arrhythmias [9]. Genetic knockout of K_ATP_ is reported to mimic the effects of pressure-overloading induced by acute aortic constriction [10]. These findings suggest that functional K_ATP_ provide adaptations to a variety of pressure-overload conditions associated with concentric cardiac hypertrophy. However, whether the K_ATP_ in eccentrically hypertrophied cardiac myocytes are functional and dysfunctional K_ATP_ contribute to heart failure in volume-overload hearts remain unclear. The present study was, therefore, designed to test the hypothesis that the K_ATP_ are dysfunctional in cardiac ventricular myocytes hypertrophied by volume-overloading.

## 2. Methods

### 2.1. Animal Preparation

Conformity statement: As discussed in the following respective sections, all the procedures conform to the Guide for the Care and Use of Laboratory Animals published by the US National Institutes of Health (NIH) publication no. 85-23, revised 1996. Adult male Sprague-Dawley rats of 200–250 g body weight were purchased from Charles Rivers (Mass, USA). The rats were allowed to recover and acquaint with their new environment upon arrival at the animal house of the Howard University College of Medicine for 1 week. The animals were kept under secure, clean, and controlled room temperature (70–74°F) with a 6:00 h to 18:00 h light cycle and were fed food and water *ad libitum*.

### 2.2. Eccentric Cardiac Hypertrophy

Rats were anaesthetized with pentobarbital sodium (30 mg/kg body weight, i.p.). A bulldog vascular clamp was placed across the aorta inferior to the renal vessels. The abdominal aorta was punctured at the union of the segment two thirds caudal to the renal artery and one third cephalic to the aortic bifurcation with an 18 gauge disposable needle. The needle was advanced into the abdominal aorta and vena cava at the point of anastomosis, thereby, shunting arterial blood into the venous system. A drop of cyanoacrylate glue was used to seal the aortic punctured point. The patency of the shunt was verified visually by swelling of the vena cava and the mixing of arterial and venous blood. As postoperative care, the rats were administered flunixin 2.5 mg/kg. The same procedure was performed on the age-matched sham rats, except for the insertion of the needle. On the experimentation day, visual inspection of the lungs did not show any signs or symptoms of pulmonary edema or pulmonary blood clots in all shunted animal used. Based on the results of our previous study using the same methods as those employed in the present one, these shunted rats appear to represent a reliable experimental model of eccentric cardiac hypertrophy [11] in a compensatory stage [12]. The compensation was evidenced by left ventricular wall thickness and stroke volume greater by approximately 40% and 56%, respectively, (*P* < 0.05) in the absence of a difference in ejection fraction 54–59% (*P* > 0.1), compared to sham-operated controls [12].

### 2.3. Isolation of Cardiomyocytes

All the reagents were purchased from Sigma Chemicals (St. Louis, Mo, USA). Double-distilled water from MilliQ system (Millipore Corporation, Mass, USA) was used to prepare all solutions. Stock buffer solution consisted of (mM): 113 NaCl, 4.7 KCl, 0.6 KH_2_PO_4_, 0.6 Na_2_HPO_4_, 1.2 MgSO_4_, 12 NaHCO_3_, 10 KHCO_3_, and 10 HEPES. Animals were injected with heparin sodium (1000 U/kg, i.p.) and anesthetized with pentobarbital sodium (40 mg/kg; i.p.), 20 min prior to the removal of the heart. After excision of the heart, it was quickly transferred to a Langendorff setup for retrograde coronary perfusion through the aorta at 10 mL/min (37°C) for an initial 5 min equilibration with a perfusion buffer consisting of (mM): 113 NaCl, 4.7 KCl, 0.6 KH_2_PO_4_, 0.6 Na_2_HPO_4_, 1.2 MgSO_4_, 12 NaHCO_3_, 10 KHCO_3_, 10 HEPES, 1.1 D-glucose, and 10.2 butanedione Monoxime. The experimental protocol consisted of continuing the retrograde perfusion of the hearts for 12 min to which 30 mL of a digestion buffer solution was added, consisting of (mg): 25 BSA (essentially fatty acid free), 25 Collagenase (type 2), and 3 Protease (Type XIV). The heart was, then, perfused for 5 min with 80 mL of a stop buffer, solution consisting of the perfusion buffer to which was added 5% fetal calf serum and 14 *μ*M CaCl_2_. The ventricles were cut, minced into the stop buffer and filtered; calcium was reintroduced up to 1.0 mM. The dissociated cardiomyocytes were, then, diluted and stored in Tyrode's solution. Freshly isolated myocytes showing no signs of blebs or round edges were used for up to 12 h.

### 2.4. Electrophysiological Studies

Whole-cell patch-clamp technique was used to study the potassium currents in the adult cardiomyocytes. Patch pipettes of 1-2 MΩ resistance were pulled from borosilicate glass capillary tubing with a 2-stage puller (David Kopf Instruments, Calif, USA). Ventricular myocytes were placed on the stage of an inverted microscope and perfused with an extracellular whole-cell K^+^ current buffer consisting of (mM): 5 KCl, 1 MgCl_2_, 140 NaCl, 10 HEPES, 10 D-glucose, 1 CaCl_2_, 0.2 CdCl_2_, and pH at 7.4. The pipette solution consisted of (mM): 130 potassium glutamate, 20 KCl, 5 EGTA, 5 NaCl, 1 MgCl_2_, 10 HEPES, and pH at 7.4. Capacitance was estimated by integrating the area of the capacitance transient following a 10 mV step voltage from a holding potential (HP) of −80 mV. The measured currents were divided by the cell capacitance in order to normalize for cell size changes between normal and hypertrophied cardiomyocytes. The cardiomyocytes were stimulated using pClamp 9.0 software (Molecular Devices, Calif, USA) connected to an Axopatch 200B amplifier through an A/D converter (Digidata1320A; Molecular Devices, Calif, USA)). The resulting ionic currents were displayed on a storage oscilloscope and on a computer for analysis with pClamp 9.0. All patch-clamp experiments were performed at room temperature (20–22°C). All solutions were filtered through a 0.22 *μ*m filter. The voltage dependency of I_KATP_ activation was studied by obtaining data for the respective current-voltage (I-V) relationships as follows: 350 ms step-voltages in 10 mV increments between −80 mV and +40 mV were applied. Steady-state currents, measured at the end of each current response, were plotted as a function of the command potential. The effects of cromakalim (100 *μ*M) in the presence and absence of glibenclamide (5 *μ*M) (Sigma-Aldrich, Mass, USA) were analyzed.

### 2.5. Statistical Analyses

The effects of pretreatment with the K_ATP_ agonist cromakalim and posttreatment with the K_ATP_ antagonist glibenclamide in ventricular cardiac myocytes from the rats subjected to volume-overloading were compared to the effects in the cardiomyocytes from the sham-operated control rats. The pA/pF values at each membrane potential were compared by repeated measures ANOVA with significance set at *P* < 0.05.

## 3. Results

The data in Table 1 support the development of hypertrophy within 3 weeks. Compared to the sham-operated controls, the volume-overloaded shunted rats exhibited significantly greater absolute and relative heart weights, using the same methods as we previously described, showing significant increments in left ventricular wall thickness and stroke volume in the absence of a difference in ejection fraction, compared to sham-operated controls [11].

**Figure 1:**
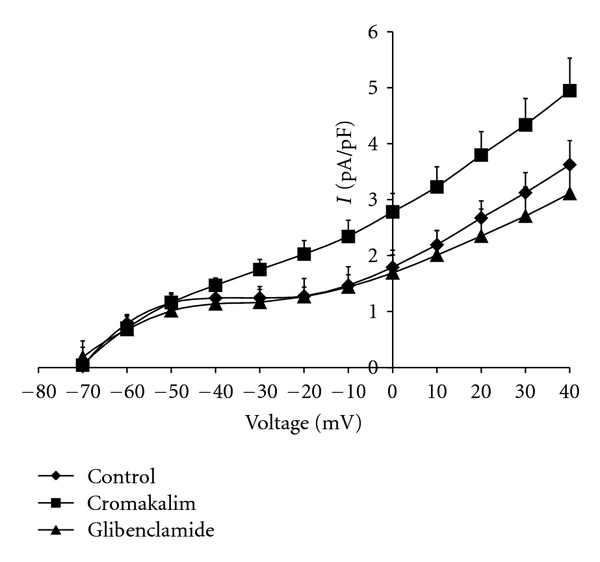
Voltage-Current Relationships in Sham-Operated Control Rats. Sarcolemmal outward potassium current (I), expressed as current density (pA/pF), in patches of ventricular myocytes from adult control rats subjected to a sham surgical operation, treatment with the ATP-sensitive potassium channel (KATP) agonist cromakalim (100 *μ*M), and post-treatment with the KATP antagonist glibenclamide (5 *μ*M). The membrane voltage was clamped at steps of 10 mV from −80 mV to +40 mV. Differences in pA/pF in the presence and absence of treatment with cromakalim, and post-treatment with glibenclamide were significant only at the voltages from −20 mV to +40 mV (*P* < 0.05).

**Figure 2:**
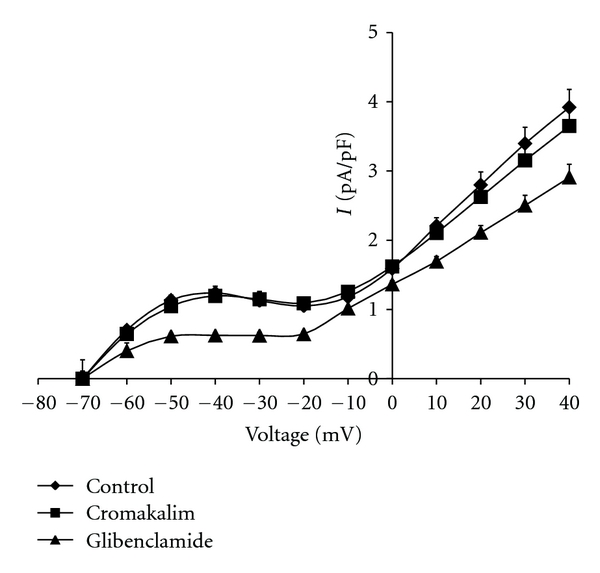
Voltage-Current Relationship in Volume-Overloaded Shunted Rats. Sarcolemmal outward potassium current (I), expressed as current density (pA/pF), in patches of ventricular myocytes from adult experimental rats subjected to a surgical operation to produce shunting of blood from the aorta to the van cava. The membrane voltage was clamped at steps of 20 mV from −80 mV to +40 mV. Differences in pA/pF in the presence and absence of treatment with cromokalim and post-treatment with glibenclamide were not significant at the voltages from −20 mV to +40 mV (*P* < 0.05).

Figures 1 and 2 depict the pA/pF at 20 mV increments of membrane potential from −80 to +40 mV demonstrating a significant difference in the basal activation level of the sarcolemmal outward potassium current density between the control and hypertrophied cardiomyocytes. Administration of the specific K_ATP_ agonist cromakalim induced significant activation of K_ATP_ above the basal level in the control cardiomyocytes at positive membrane potentials, with the greatest difference observed at positive membrane potentials. This activation was effectively blocked by post-treatment with the specific K_ATP_ antagonist glibenclamide which restored the current density to the control level. Treatment of the hypertrophied cardiomyocytes with cromakalim resulted in outward current density that was not significantly different than that of the control and posttreatment with glibenclamide had no effect (Figure 2). These administrations of cromakalim and glibenclamide to control and hypertrophied myocytes did not produce any significant changes in the slope conductance g_KATP_ (shunts 53.7 ± 6.0 nS/pF versus controls 52.1 ± 13.6 nS/pF).

**Figure 3:**
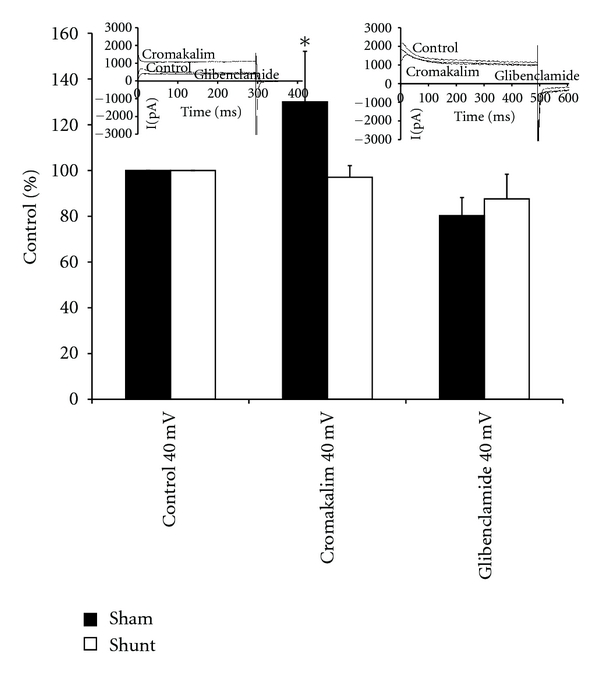
Comparison of Effects of Cromakalim and Glibenclamide. Bars represent the sarcolemmal outward potassium current density amplitude, expressed as percentages of the control values of ventricular myocytes from control, sham-operated and shunted, volume-overloaded rats, measured at the clamp voltage of +40 mV under conditions of pretreatment with cromokalim and post-treatment with glibenclamide. Asterisk (∗) indicates statistically significant difference from control and shunt at *P* < 0.05. Inserts are raw data recordings showing the amplitudes of outward potassium currents (I), expressed in pA, acquired at a membrane clamp voltage of +40 mV during the control condition, pretreatment with cromokalim and post-treatment with glibenclamide; left insert from a sham-operated control and right insert from a shunted volume-overloaded rat.

Figure 3 presents comparisons of the current density at +40 mV as percentages of the control values. The cromakalim treatment increased current density significantly above the control value in the control cardiomyocytes but not in the hypertrophied myocytes. The glibenclamide posttreatments were associated with maintenance of the control values. 

## 4. Discussion

### 4.1. K_ATP_ Antagonism, Cardiac Hypertrophy, and Heart Failure

Dysfunctional K_ATP_ can contribute to cardiac failure by various mechanisms that can be studied by blocking them with the specific antagonist glibenclamide [13]. Blockade of K_ATP_ with glibenclamide has been shown to mimic the decompensating effects of pressure-overloading induced by acute aortic constriction observed in knockout mice lacking the Kir6.2 pore of K_ATP_ [10]. This finding suggests a requirement for functional K_ATP_ to induce compensatory cardiac myocyte hypertrophy and protect against ventricular dilatation and heart failure. Blockade of K_ATP_ with glibenclamide is also reported to abolish the cardiac antihypertrophic effects and postinfarction remodeling of border zone myocardium mediated by activated K_ATP_ [14, 15]. In addition, glibenclamide appears to inhibit the hypertrophy associated with myocardial remodeling following hydrogen sulfide treatment of spontaneously hypertensive rats [16]. Inhibition of a Ca^++^-activated nonselective transient receptor membrane potential (TRPM4) channel, which is overexpressed in various experimental models of cardiac hypertrophy, is also attributed to antagonism of K_ATP_ by glibenclamide [17]. These findings suggest that K_ATP_ dysfunction is likely to be a common pathophysiological feature of a diverse group of conditions associated with cardiac hypertrophy and heart failure. 

### 4.2. Actions of the K_ATP_ Antagonist Glibenclamide

Glibenclamide did reverse the antihypertrophic effects of sarcolemmal and mitochondrial K_ATP_ agonists following cardiac myocyte hypertrophy associated with activation of the adenosine receptors in neonatal rat ventricular myocytes [18]. Glibenclamide has also effectively antagonized the anti-hypertrophic effects of diazoxide [19], pravastatin [20], and KR-31378 [21] on cardiac myocytes in various experimental models of hypertension. However, in a Langendorff preparation using constant perfusion pressure, glibenclamide failed to antagonize the hypertrophic effects of carbon monoxide [22]. These findings suggest an essential difference between carbon monoxide and other models of cardiac hypertrophy that may be explainable by reports that carbon monoxide exerts protective effects on cardiomyocytes via activation of mitochondrial K_ATP_ [23]. Moreover, sulfonylurea binding sites of the mitochondrial K_ATP_ of cardiomyocytes may either be lacking or of insufficient sensitivity to respond to glibenclamide.

### 4.3. Actions of the K_ATP_ Agonist Cromakalim

Although we found that the hypertrophied ventricular cardiomyocytes from volume-overloaded animals were unresponsive to the K_ATP_ agonist cromakalim, the cardiomyocytes from the sham-operated control animals did respond to cromakalim. Cromakalim-induced increments in the sarcolemmal current density are likely to shorten the ventricular action potential. Shortening the ventricular action potential should make the heart more susceptible to tachycardia, one of the hallmarks of cardiac stress. In addition, this shortening represents an adaptation that is suited for ameliorating the adverse effects of decreased contractility and the potential for low cardiac output associated with cardiac hypertrophy and heart failure. Indeed, the main functions of K_ATP_ seem to be as responders to cardiac stress [24] and shortening the duration of the cardiac action potential should decrease myocyte Ca^++^ influx and force of contraction, and, consequently, the requirement for ATP which is in short supply in hypertrophic cardiac myocytes [25]. Hypertrophic cardiac myocytes are also reported to exhibit changes in the cAMP level and in *β*-adrenergic receptor activity [26]. cAMP phosphodiesterase inhibition is reported to be useful for treating cardiac hypertrophy [27], thereby suggesting that high cAMP activity may inhibit cardiac myocyte hypertrophy. Decreased expression of cAMP phosphodiesterase, which should increase the myocyte cAMP level, appears to modulate the sensitivity of the G-protein coupled *β*-adrenergic receptors in hypertrophic cardiac myocytes [28]. Moreover, a hyperpolarization-activated, cyclic nucleotide-gated current, also thought to be modulated by G-protein coupled receptors, is overexpressed in hypertrophied cardiac myocytes [29]. Therefore, the combined effects of K_ATP_-mediated shortening of the action potential and refractory period should facilitate *β*-adrenergic modulation of the heart rate as an orchestrated response, likely mediated by G-protein-coupled adrenergic receptors, thereby, maintaining cardiac output during cardiac myocyte hypertrophy. That, in the present study, hypertrophied ventricular cardiomyocytes from volume-overloaded animals were found to be unresponsive to the K_ATP_ agonist cromakalim suggest that these myocytes might also be unresponsive to catecholamines. Indeed, cromakalim is reported to mimic the effects of adenosine that make rat ventricular myocytes insensitive to alpha-adrenergic agonists by a G(i) protein-dependent mechanism [30].

### 4.4. Role of K_ATP_ in Cardiac Hypertrophy and Heart Failure Therapies

The apparent insensitivity of K_ATP_ channels to volume-overload that we describe in rat hearts suggests that some of the therapeutic approaches already in use for ameliorating the adverse consequences of K_ATP_ channel dysfunction may, in some subsets of patients, be ineffective. Current approaches include activation of K_ATP_ channels by the vasodilator agents nicorandil or pinacidil that is reported to attenuate the ventricular remodeling associated with myocardial infarction in rats [15] and by iptakalim for protecting the endothelium in pressure-overloaded rats [31]. Future immunologic therapies will, no doubt, be based on emergent evidence that tumor necrosis factor (TNF-alpha) is an important humoral mediator of K_ATP_ channel remodeling in cardiomyocyte hypertrophy and heart failure [32]. A more complex therapeutic approach involves embryonic stem cell administration to hearts affected by dilated cardiomyopathy induced by K_ATP_ channel knockout that is reported to produce a proteome profile indicative of amelioration of cardiac hypertrophy and heart failure [33]. For example, a potentially useful experimental animal model of human dilated cardiomyopathy, a progressive organ dysfunction syndrome refractory to conventional therapies and linked to mutations in cardiac K_ATP_ channel subunits, appears to have been produced in a Kir6.2-knockout mouse model [34]. The absence of functional K_ATP_ channels appears to have been overcome by epicardial delivery of embryonic stem cells to the left ventricle reported to reverse the electromechanical manifestations of systolic dysfunction, as well as the maladaptive remodeling [34].

## 5. Conclusions

In summary, using aortocaval shunting to produce a rat model of volume-overload, previously shown to produce compensated eccentric cardiac hypertrophy [11, 12], the K_ATP_ antagonist glibenclamide had no significant effect on K_ATP_ currents. This finding is in sharp contrast to glibenclamide having the expected effect of counteracting cromakalim-induced increments in K_ATP_ currents in our control cardiomyocytes and of mimicking the adverse effects of cardiomyocyte action potential prolongation, calcium overload, and ATP depletion reported in K_ATP_ Kir 6.2 pore knockout pressure-overloaded mice [10]. The findings of the present study suggest the hypothesis that what seems to be a marked insensitivity of sarcolemmal potassium current to K_ATP_ antagonism by glibenclamide may contribute to greater morbidity and mortality associated with volume-overload (e.g., aortic regurgitation) than with pressure overload (e.g., aortic stenosis) in humans. This hypothesis appears to be supported by studies showing that aortic regurgitation produces symptoms and/or left ventricular systolic dysfunction at the average rate of 4.3% per year with sudden death reported in 7 of 593 patients reviewed, about 1% [35]. In contrast to patients with aortic regurgitation, sudden death is considered to be a rare occurrence in patients with aortic stenosis, estimated at less than 1% per year [35]. The importance of limiting the progression of left ventricular hypertrophy to ventricular dilatation is demonstrated by serial longitudinal studies showing that patients with aortic regurgitation and end-systolic dimensions greater than 50 mm exhibited death, symptoms, and/or left ventricular dysfunction at an average rate of 19% per year, but only 6% per year in patients with left ventricular dimensions 40 mm−50 mm and zero in patients with left ventricular dimensions less than 40 mm [36]. These findings suggest that, in a subset of patients with aortic regurgitation, the adaptive effects of cardiomyocyte hypertrophy may be limited, perhaps by dysfunctional K_ATP_, akin to the volume-overloaded rats in the present study exhibiting insensitivity to the K_ATP_ agonist and antagonist cromakalim and glibenclamide.

## Figures and Tables

**Table 1:** Heart weights of sham-operated control and shunted volume-overloaded rats.

	Heart weight (mg)	Relative heart weight (mg/100 g body weight)
Sham	1191 ± 3	327 ± 1
Shunt	2307 ± 52*	433 ± 6*

*P < 0.05, sham versus shunt.

## References

[1] Kannel WB (2000). Incidence and epidemiology of heart failure. *Heart Failure Reviews*.

[2] Turina J, Hess O, Sepulcri F, Krayenbuehl HP (1987). Spontaneous course of aortic valve disease. *European Heart Journal*.

[3] Flagg TP, Enkvetchakul D, Koster JC, Nichols CG (2010). Muscle K_ATP_ channels: recent insights to energy sensing and myoprotection. *Physiological Reviews*.

[4] Li GR, Dong MQ (2010). Pharmacology of cardiac potassium channels. *Advances in Pharmacology*.

[5] Kolár F, Neckár J, Ostádal B (2005). MCC-134, a blocker of mitochondrial and opener of sarcolemmal ATP-sensitive K^+^ channels, abrogates cardioprotective effects of chronic hypoxia. *Physiological Research*.

[6] Han J, Kim N, Joo H, Kim E (2002). Ketamine abolishes ischemic preconditioning through inhibition of K_ATP_ channels in rabbit hearts. *American Journal of Physiology*.

[7] Tsutsumi S, Kushiku K, Kuwahara T, Tokunaga R, Furukawa T (1995). Ionotropic mechanisms involved in postsynaptic inhibition by the endothelins of ganglionic transmission in dog cardiac sympathetic ganglia. *Journal of Cardiovascular Pharmacology*.

[8] Kakkar R, Ye B, Stoller DA (2006). Spontaneous coronary vasospasm in K_ATP_ mutant mice arises from a smooth muscle-extrinsic process. *Circulation Research*.

[9] Quindry JC, Schreiber L, Hosick P, Wrieden J, Irwin JM, Hoyt E (2010). Mitochondrial K_ATP_ channel inhibition blunts arrhythmia protection in ischemic exercised hearts. *American Journal of Physiology*.

[10] Yamada S, Kane GC, Behfar A (2006). Protection conferred by myocardial ATP-sensitive K^+^ channels in pressure overload-induced congestive heart failure revealed in KCNJ11 Kir6.2-null mutant. *Journal of Physiology*.

[11] Abassi Z, Goltsman I, Karram T, Winaver J, Hoffman A (2011). Aortocaval fistula in rat: a unique model of volume-overload congestive heart failure and cardiac hypertrophy. *Journal of Biomedicine and Biotechnology*.

[12] Teos LY, Zhao A, Alvin Z, Laurence GG, Li C, Haddad GE (2008). Basal and IGF-I-dependent regulation of potassium channels by MAP kinases and PI3-kinase during eccentric cardiac hypertrophy. *American Journal of Physiology*.

[13] Ripoll C, Lederer WJ, Nichols CG (1993). On the mechanism of inhibition of K_ATP_ channels by glibenclamide in rat ventricular myocytes. *Journal of Cardiovascular Electrophysiology*.

[14] Lee TM, Lin MS, Tsai CH, Chang NC (2007). Effects of pravastatin on ventricular remodeling by activation of myocardial K_ATP_ channels in infarcted rats: role of70-kDa S6 kinase. *Basic Research in Cardiology*.

[15] Lee TM, Lin MS, Chang NC (2008). Effect of ATP-sensitive potassium channel agonists on ventricular remodeling in healed rat infarcts. *Journal of the American College of Cardiology*.

[16] Shi YX, Chen Y, Zhu YZ (2007). Chronic sodium hydrosulfide treatment decreases medial thickening of intramyocardial coronary arterioles, interstitial fibrosis, and ROS production in spontaneously hypertensive rats. *American Journal of Physiology*.

[17] Demion M, Bois P, Launay P, Guinamard R (2007). TRPM4, a Ca^2+^-activated nonselective cation channel in mouse sino-atrial node cells. *Cardiovascular Research*.

[18] Xia Y, Javadov S, Gan TX, Pang T, Cook MA, Karmazyn M (2007). Distinct K_ATP_ channels mediate the antihypertrophic effects of adenosine receptor activation in neonatal rat ventricular myocytes. *Journal of Pharmacology and Experimental Therapeutics*.

[19] Xia Y, Rajapurohitam V, Cook MA, Karmazyn M (2004). Inhibition of phenylephrine induced hypertrophy in rat neonatal cardiomyocytes by the mitochondrial K_ATP_ channel opener diazoxide. *Journal of Molecular and Cellular Cardiology*.

[20] Lee TM, Lin MS, Tsai CH, Chang NC (2006). Effect of pravastatin on left ventricular mass in the two-kidney, one-clip hypertensive rats. *American Journal of Physiology*.

[21] Hwang GS, Oh KS, Koo HN, Seo HW, You KH, Lee BH (2006). Effects of KR-31378, a novel ATP-sensitive potassium channel activator, on hypertrophy of H9c2 cells and on cardiac dysfunction in rats with congestive heart failure. *European Journal of Pharmacology*.

[22] Barbé C, Rochetaing A, Kreher P (2002). Mechanisms underlying the coronary vasodilation in the isolated perfused hearts of rats submitted to one week of high carbon monoxide exposure in vivo. *Inhalation Toxicology*.

[23] Soni H, Patel P, Rath AC, Jain M, Mehta AA (2010). Cardioprotective effect with carbon monoxide releasing molecule-2 (CORM-2) in isolated perfused rat heart: role of coronary endothelium and underlying mechanism. *Vascular Pharmacology*.

[24] Kane GC, Liu XK, Yamada S, Olson TM, Terzic A (2005). Cardiac K_ATP_ channels in health and disease. *Journal of Molecular and Cellular Cardiology*.

[25] Dolinsky VW, Dyck JRB (2006). Role of AMP-activated protein kinase in healthy and diseased hearts. *American Journal of Physiology*.

[26] Filipeanu CM, Zhou F, Lam ML, Kerut KE, Claycomb WC, Wu G (2006). Enhancement of the recycling and activation of *β*-adrenergic receptor by Rab4 GTPase in cardiac myocytes. *Journal of Biological Chemistry*.

[27] Osadchii OE (2007). Myocardial phosphodiesterases and regulation of cardiac contractility in health and cardiac disease. *Cardiovascular Drugs and Therapy*.

[28] Abi-Gerges A, Richter W, Lefebvre F (2009). Decreased expression and activity of cAMP phosphodiesterases in cardiac hypertrophy and its impact on *β*-Adrenergic cAMP signals. *Circulation Research*.

[29] Stillitano F, Sartiani L, DePaoli P, Mugelli A, Cerbai E (2008). Expression of the hyperpolarization-activated current, *I*
_f_, in cultured adult rat ventricular cardiomyocytes and its modulation by hypertrophic factors. *Pharmacological Research*.

[30] Hoque N, Cook MA, Karamzyn M (2000). Inhibition of *α*
_1_-adrenergic-mediated responses in rat ventricular myocytes by adenosine A_1_ receptor activation: role of the K_ATP_ channel. *Journal of Pharmacology and Experimental Therapeutics*.

[31] Gao S, Long CL, Wang RH, Wang H (2009). K_ATP_ activation prevents progression of cardiac hypertrophy to failure induced by pressure overload via protecting endothelial function. *Cardiovascular Research*.

[32] Tavares NI, Philip-Couderc P, Baertschi AJ, Lerch R, Montessuit C (2009). Angiotensin II and tumour necrosis factor *α* as mediators of ATP-dependent potassium channel remodelling in post-infarction heart failure. *Cardiovascular Research*.

[33] Arrell DK, Lindor JZ, Yamada S, Terzic A (2011). K_ATP_ channel-dependent metaboproteome decoded: systems approaches to heart failure prediction, diagnosis, and therapy. *Cardiovascular Research*.

[34] Yamada S, Nelson TJ, Crespo-Diaz RJ (2008). Embryonic stem cell therapy of heart failure in genetic cardiomyopathy. *Stem Cells*.

[35] Bonow RO, Carabello BA, Chatterjee K (2008). 2008 focused update incorporated into the ACC/AHA 2006 guidelines for the management of patients with valvular heart disease: a report of the American college of cardiology/American heart association task force on practice guidelines (writing committee to revise the 1998 guidelines for the management of patients with valvular heart disease): endorsed by the society of cardiovascular. *Circulation*.

[36] Bonow RO, Lakatos E, Maron BJ, Epstein SE (1991). Serial long-term assessment of the natural history of asymptomatic patients with chronic aortic regurgitation and normal left ventricular systolic function. *Circulation*.

